# Pilot Study for OCT Guided Design and Fit of a Prosthetic Device for Treatment of Corneal Disease

**DOI:** 10.1155/2012/812034

**Published:** 2012-12-18

**Authors:** Hong-Gam T. Le, Maolong Tang, Ryan Ridges, David Huang, Deborah S. Jacobs

**Affiliations:** ^1^Boston Foundation for Sight, 464 Hillside Avenue, Suite 205, Needham, MA 02494, USA; ^2^Center for Ophthalmic Optics and Lasers, Casey Eye Institute, Oregon University of Health & Science, Portland, OR, USA; ^3^Department of Ophthalmology, Harvard Medical School, Boston, MA, USA

## Abstract

*Purpose*. To assess optical coherence tomography (OCT) for guiding design and fit of a prosthetic device for corneal disease. *Methods*. A prototype time domain OCT scanner was used to image the anterior segment of patients fitted with large diameter (18.5–20 mm) prosthetic devices for corneal disease. OCT images were processed and analyzed to characterize corneal diameter, corneal sagittal height, scleral sagittal height, scleral toricity, and alignment of device. Within-subject variance of OCT-measured parameters was evaluated. OCT-measured parameters were compared with device parameters for each eye fitted. OCT image correspondence with ocular alignment and clinical fit was assessed. *Results*. Six eyes in 5 patients were studied. OCT measurement of corneal diameter (coefficient of variation, CV = 0.76%), cornea sagittal height (CV = 2.06%), and scleral sagittal height (CV = 3.39%) is highly repeatable within each subject. OCT image-derived measurements reveal strong correlation between corneal sagittal height and device corneal height (*r* = 0.975) and modest correlation between scleral and on-eye device toricity (*r* = 0.581). Qualitative assessment of a fitted device on OCT montages reveals correspondence with slit lamp images and clinical assessment of fit. *Conclusions*. OCT imaging of the anterior segment is suitable for custom design and fit of large diameter (18.5–20 mm) prosthetic devices used in the treatment of corneal disease.

## 1. Introduction

**Figure 1:**
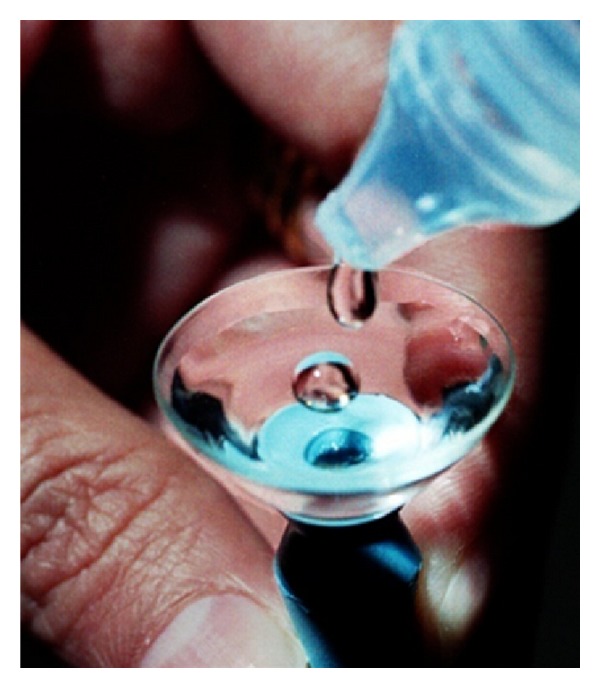
A PROSE device being filled with sterile saline solution.

**Figure 2:**
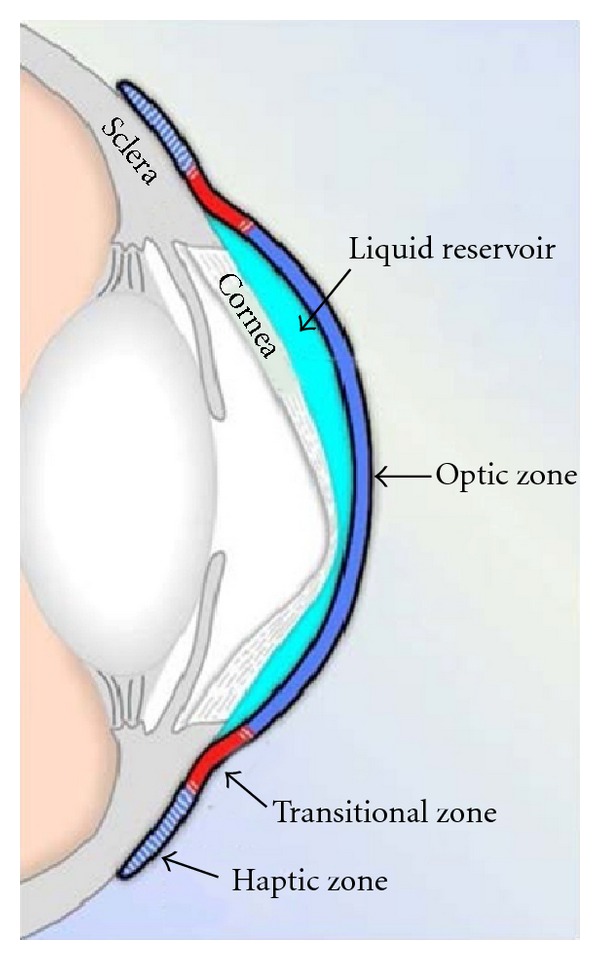
Schematic diagram of a PROSE device fitted to an eye with corneal ectasia.

**Figure 3:**
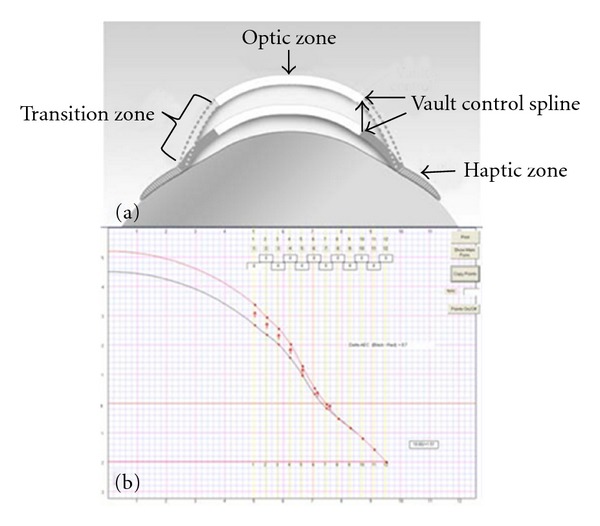
(a) Schematic diagram of a PROSE device illustrating how transitional zone is used to allow for vault independent of base curve. (b) Screen shot of the CAD/CAM system used for PROSE device customization.

Prosthetic Replacement of the Ocular Surface Ecosystem (PROSE) treatment is an evolving approach to complex corneal disease developed at Boston Foundation for Sight (Needham, MA, USA) [1, 2]. PROSE treatment uses FDA-approved, custom designed, rigid gas-permeable prosthetic devices that vault the cornea and rest entirely on the sclera. A PROSE device is filled with sterile saline prior to application to the eye (Figure 1). A customized PROSE device restores function to the entire ocular surface system by creating a transparent, smooth optical surface over the irregular, damaged or diseased cornea, by establishing an expanded oxygenated tear reservoir, and by protecting the corneal surface from adverse effects of the environment and lid abnormalities. A PROSE device is characterized by a central *optic zone*, a *transitional zone*, and a peripheral *haptic zone* (Figure 2). The optic zone and the transitional zone can be customized to neutralize refractive error and address anatomic anomalies of the corneal topography, respectively. The contours of the haptic zone can be specified separately for “fit” based on clinical criteria. The mathematical description of these contours is specified by software that utilizes spline functions and is integral to the Design to Fit (DTF) web-based CAD/CAM system (Figure 3) that links the clinician who custom designs and fits these devices to a manufacturing lathe. The spline-controlled zones allow for the vault of the device to be specified independent of the base curve of the optic zone. It is this design flexibility and the very high degree of customization of a PROSE device compared with a conventional scleral lens that ensures ability to achieve good fit and high level of prosthetic function [3, 4]. 

Two additional critical design features of a PROSE device are that it does not touch the cornea and that there is virtually no movement of the weight-bearing haptic on the sclera. The latter is accomplished by precise alignment with the sclera. Current practice for PROSE treatment is that of the “diagnostic” (versus “empiric”) approach to contact lens fitting. In the “diagnostic” approach to contact lens fitting, a trial device is placed on the eye, and subsequent modification of lens parameters is based on clinical observation. This approach differs from the “empiric” approach of contact lens fitting, used typically for RGP corneal contact lens fitting, in which a parameter of an initial trial lens is selected empirically to be steeper than K, with K referring to keratometry reading or a simulated value taken from video-keratography. 

In PROSE treatment, the fit of a trial device is based on clinical assessment of numerous parameters over increasing time intervals. An apparently inadequate device is removed immediately after application and assessment. A satisfactory device is reassessed after 1 hour, 3 hours, and then 6 hours, at which time specific parameters are modified by selection of another device from an inventory of trial devices or by design and manufacture of a more appropriate device. The assessment has begun again. Optics are customized based on overrefraction and incorporated into the front surface of any device cut de novo. This iterative, custom design process is resource-intensive, requiring trial and/or production of numerous devices per eye. The fitting process combined with training the patient in device application and removal requires typically 4–10 days in the clinician's office with visits spread out over time based on proximity to the single site of manufacturing, and on patient and clinician scheduling preferences. Although PROSE treatment is cost-effective by contemporary standards [5], the resources required limit wider utilization and wider therapeutic impact. 

OCT is a noncontact imaging technology that provides detailed cross-sectional images of internal structures in biological tissues [6]. High-resolution OCT of the anterior segment of the eye can provide data on the shape of the sclera which is not available via keratometry or videokeratography, and the morphometric tools are typically used to fit corneal contact lenses [7]. These technologies require specular reflection and have been shown not to correlate with fit of scleral lens [8]. There is a report on the use of OCT to fit scleral lenses but contour analysis was limited to 15 mm diameter and the diameter of the lenses fitted was not reported [9]. Corneoscleral topography as evaluated by OCT has been shown to provide insight into soft contact lens fit dynamics [10], but relevance to PROSE treatment is limited. By definition, fit of a PROSE device is not dynamic; furthermore soft contact lenses are typically diameter of 13–15 mm, whereas PROSE devices range from 17.5 to 24 mm diameter. 

The objective of this pilot study was to investigate if OCT image-derived measurements of the cornea and sclera out to 17 mm correlate with parameters of fit for patients fitted with a PROSE device (18.5–20 mm) using a time domain OCT prototype. Once such correlation is established, further protocols for image-guided design and fit can be developed, with the goal of reducing resources required to customize a device for each eye. 

## 2. Methods

### 2.1. Study Design

This clinical prospective study was conducted at Boston Foundation for Sight in Needham, MA, USA. The research protocol was approved by the New England Institutional Review Board and was performed in accordance with the Declaration of Helsinki. Written informed consent was obtained from each subject after explanation of the nature and possible consequences of the study.

### 2.2. Study Population

Subjects were recruited from patients undergoing fitting of a PROSE device at Boston Foundation for Sight, or patients previously fitted returning for a routinely scheduled visit. PROSE devices of 18.5–20 mm diameter were fitted according to the standard iterative approach using trial devices and custom modification of device contours based on clinical findings. 

### 2.3. OCT Imaging

A prototype time domain 1310 nm wavelength anterior segment OCT scanner (Optovue, Inc., Freemont, CA) which operates at 2000 axial scans per second and axial resolution of 17 *μ*m was used to image the anterior segment of 6 eyes in 5 patients. OCT imaging using a combination of radial and circular scans in primary and deviated gaze was obtained of each eye before and while wearing a fitted PROSE device. Manual lid retraction was required during image acquisition. Three scans were obtained during each session.

### 2.4. Slit Lamp Imaging

Slit lamp photographs of each eye in a fitted device were taken to document device orientation and clinical features of fit. 

### 2.5. Analysis of OCT Measurements

**Figure 4:**
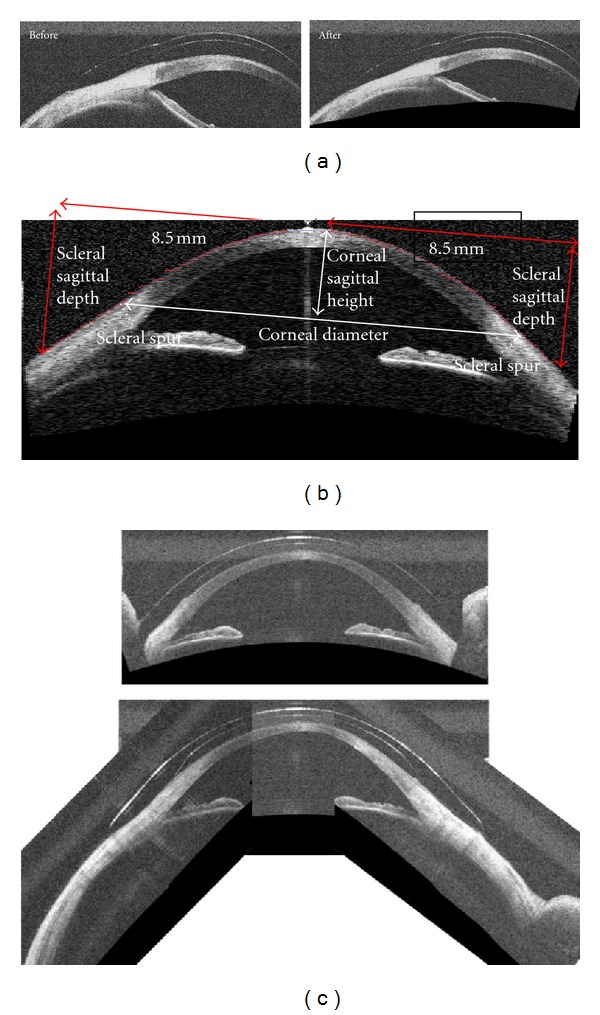
(a) OCT scans before and after dewarping. (b) A normal cornea imaged with a time-domain anterior segment OCT system. The image dimension is 18 mm and consists of 256 axial scans acquired in 0.128 second. (c) Vertical scan with lid artifact (red arrows) and montage of images obtained in deviated gaze to create image of entire anterior globe contour absent lid artifact.

**Figure 5:**
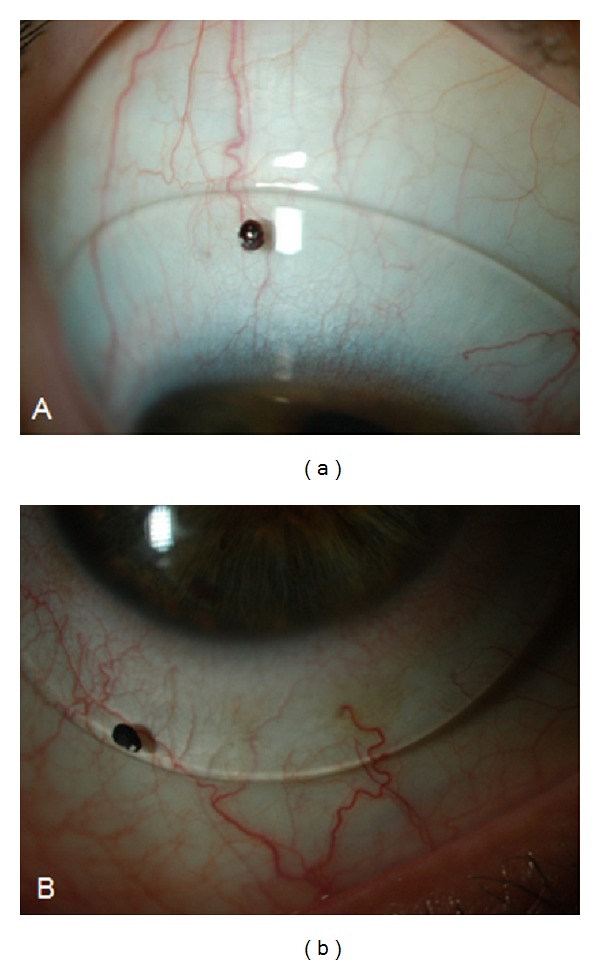
Slit lamp photos showing device orientation used to define sampling axis of device profile to test for correlation with scleral toricity derived from OCT image. (a) Eye #3: 0 degrees of rotation. (b) Eye #6: 30 degrees of rotation.

OCT image processing software was developed to de-warp scans (Figure 4(a)) and to generate elevation profiles (Figure 4(b)). Ultrawide montages were created incorporating scans taken in extremes of gaze (Figure 4(c)). These were analyzed to obtain measurements of corneal diameter, corneal sagittal height, scleral sagittal height, and scleral toricity at the cardinal axes. As shown in Figure 4(b), *corneal diameter* is defined as the distance between the scleral spurs in the OCT image derived from scans along the horizontal and vertical meridians. *Corneal sagittal height* is defined as the perpendicular distance from corneal apex to the chord of corneal diameter. *Device corneal height* is defined as the perpendicular distance from the on-eye device posterior surface to the chord of corneal diameter. *Scleral sagittal height* is defined as the perpendicular distance from a 17 mm chord, parallel to the corneal apex, to the sclera. *Device sagittal height* is defined as the perpendicular distance from a 17 mm chord to the device posterior surface, extracted from device profiles specified by DTF. *Scleral toricity* is defined as the difference in average scleral sagittal height between the horizontal (0° & 90°) and vertical (90° & 270°) meridians. *Device toricity* is defined as the difference in average device sagittal height between the horizontal (0° & 90°) and vertical (90° & 270°) meridians, extracted from device profiles specified by DTF. The device sagittal heights were adjusted for rotation observed clinically (Figure 5). 

### 2.6. Statistical Analysis

Pooled standard deviation of repeat measurements was used to evaluate repeatability. Paired *t*-test and Bland-Altman plots [11] were used to evaluate the agreement between OCT measurements and device parameters. Statistical analysis was performed using Microsoft Excel.

## 3. Results

### 3.1. Patient Characteristics

**Figure 6:**
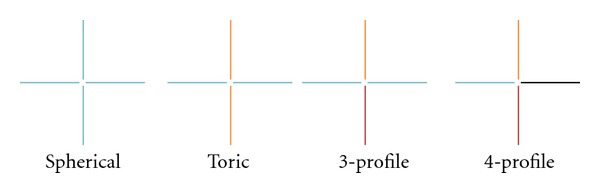
Diagrammatic representation of various radially symmetric and asymmetric haptic types. Each color represents a unique contour.

Six eyes of 5 subjects were enrolled and completed the study. The average age of subjects was 55 (range: 49–61) and the sample comprised 2 males and 3 females. Four out of 5 subjects had primary diagnosis of keratoconus, the remaining subject had dry eye syndrome. One eye was fit with a device having a rotationally symmetric haptic; the others required customized asymmetric haptic types represented diagrammatically in Figure 6. Devices were ranged from 18.5 to 20 mm diameter. Characteristics of device fitted for each eye studied are presented in Table 1. 

### 3.2. Within-Subject Variance of OCT-Measured Parameters

We evaluated the reliability of the measurement of the OCT scanner by assessing within-subject variance of OCT-measured corneal diameter, corneal sagittal height, scleral sagittal height, and scleral toricity. The mean value, pooled standard deviation, and coefficient of variation for each of the anterior segment dimensions are presented in Table 2. Within each subject, OCT measurement of corneal diameter, corneal sagittal height, and scleral sagittal height are highly repeatable. 

### 3.3. Correlation between Corneal Sagittal Height and Device Corneal Height

**Figure 7:**
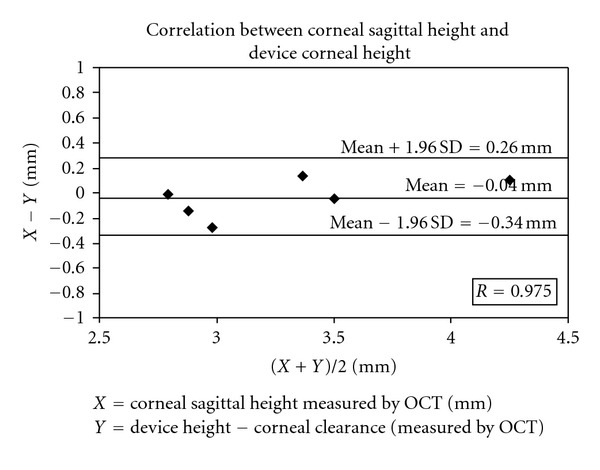
Bland-Altman plot of the correlation between OCT-measured corneal sagittal height and device corneal height.

Corneal sagittal height was measured from an OCT image of the eye not wearing a device. Device corneal height was measured from an image of the eye wearing a device. The interclass correlation coefficient was 0.975 and the 95% confidence interval is 0.959–0.991. The agreement between corneal sagittal height and device corneal height is shown in Figure 7. 

### 3.4. Correlation between Scleral and Device Toricity

**Figure 8:**
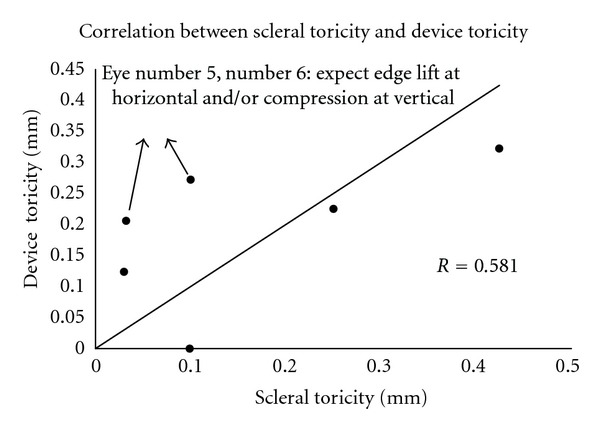
Correlation between scleral toricity and device toricity.

Scleral toricity was measured from OCT image of each eye. Device toricity was calculated based on DTF design parameters adjusted for the clinically observed rotation. The interclass correlation coefficient was 0.581 with a 95% confidence interval of 0.436–0.726. A scatter plot shows the relationship between scleral toricity and device toricity (Figure 8). The straight line represents perfect correlation. Notice that values for Eye #5 and Eye #6 are most deviated from expected values. The differences could be due to unsatisfactory features of fit such as “haptic compression” or “edge lift,” and/or failure to accurately adjust for device rotation on the eye. 

### 3.5. Qualitative Correlation between OCT Image and Satisfactory and Unsatisfactory Haptic Fit

**Figure 9:**
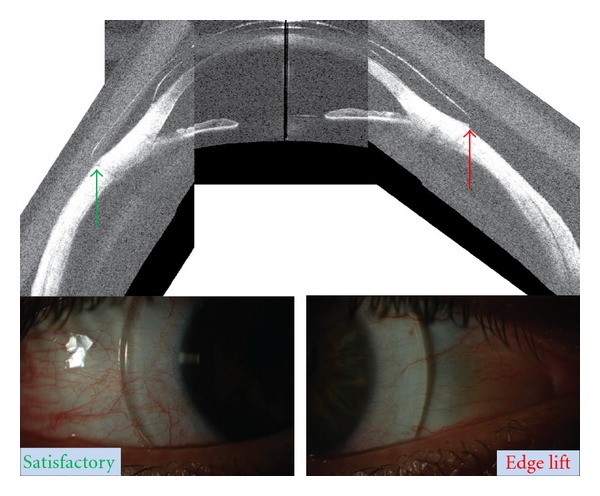
Horizontal montage (TD-OCT) and slit lamp photographs of Eye #6. Green arrow indicates satisfactory fit temporally with correlating slit lamp image below; red arrow points to edge lift which is marked by shadow just peripheral to the nasal edge of the haptic in the slit lamp image below.

Subjective assessment of fitted devices on OCT images revealed correspondence with slit lamp images revealing satisfactory alignment of the haptic over the limbus and sclera. Features of unsatisfactory fit, such as edge lift and compression, could also be detected on OCT images. Analysis of correlation between scleral and device toricity for Eye #6 suggests edge lift at the horizontal meridian and/or compression at the vertical meridian (Figure 8). Slit lamp photograph and horizontal OCT montage suggest edge lift nasally, thereby confirming the image-derived analysis (Figure 9). Similar agreement was observed in Eye #5 between the slit lamp photograph and OCT image.

## 4. Discussion

 PROSE devices are currently fitted using a diagnostic approach, with clinician's choice of a first lens based not on specific measurement, but on a subjective clinical assessment of the diameter and vault likely to be required. Refinement of contours is then carried out in an iterative process in which incremental changes are sequentially evaluated on the eye. The resources required to custom design and fit these devices are an impediment to wider utilization. Image-guided fitting could be an important step toward an automated approach to customization of these devices, leading to a more efficient fit, a better fit, or both. Images could form a basis for empiric, diagnostic, or combination approaches to design and fitting. The advances developed for PROSE treatment have potential to extend to contact lens fitting in general, to the fitting of large diameter contact lenses, and to the design of other ophthalmic medical devices that bear anatomic relation to the anterior segment of the eye. 

This pilot study, using objective and quantitative assessment of agreement between OCT-derived measurements of the cornea and sclera, and parameters of a fitted PROSE device, demonstrates that high-resolution anterior segment OCT can provide profiles and indices useful for the custom design and fit of a large diameter prosthetic device for corneal disease. This pilot study reveals high repeatability of OCT measurement of anterior segment dimensions, strong correlation between corneal sagittal height and device corneal height (*r* = 0.975), and some correlation between scleral toricity and device toricity (*r* = 0.581) in this small sample. Furthermore, qualitative assessment of the fitted devices on OCT montages revealed correspondence with clinical assessment and slit lamp images of haptic fit in pathological eyes. These findings support the hypothesis that OCT is suitable to guide the design and fit of a prosthetic device for treatment of corneal disease. We found only modest correlation between scleral toricity and device toricity; this may be due to inconsistencies in operator-dependent image acquisition, rotational movement of device on eye, failure to accurately adjust for device rotation, and variations in fit. Our sample size was small, further limiting statistical power to detect correlation of scleral and device toricity. 

Another limitation of this study is that the OCT scanner used in this study was a time-domain prototype, limited in both spatial resolution and acquisition speed. The accuracy of OCT imaging is limited by eye movements during scan, which may lead to error in image-derived measurements. Fourier-domain (FD-OCT), which allows for better image quality and higher imaging speed in comparison to time-domain (TD-OCT), [12, 13] should improve the accuracy of measurements. While PROSE devices studied here were of diameter 18.5–20 mm, our OCT measurement was limited to 17 mm with the system used in this study. A wider scan, currently in development, will allow gathering more data relevant to the fit of the haptic and may eliminate the need for the use of montages

A constraint we encountered was that the superior and inferior limbus and sclera are covered by the eyelids in primary gaze. Our solution was to create montages of images acquired in deviated gaze. Globe contour may vary with action of the extraocular muscles introducing error, as may the creation of montages. The use of a lid speculum is an alternative that we explored; subjects found this uncomfortable despite the use of topical anesthetic and trial of various speculum sizes and designs, preferring manual lid retraction, furthermore the superior limbus was not always adequately exposed with the speculum. A wider scan will not eliminate the obscuration of superior and inferior contours by the lids. It may be that systems for standard lid retraction, for creation of montages for analysis, and for interpretation of findings on deviated gaze will remain important.

This pilot study represents an initial investigation of the suitability of an OCT derived image-guided approach to the design and fit of a prosthetic device for treatment of corneal disease. This study, limited by sample size, finds that the fit of a PROSE device correlates modestly with globe surface contours detected by OCT. In theory, data on corneal, limbal and scleral contours could be “plugged into” the CAD-CAM platform on which PROSE devices are designed, streamlining or bypassing the diagnostic, iterative process by which device and scleral contours are matched in the current customization process. OCT could provide an empiric shortcut to fitting. Despite the resource requirements that are an impediment to wider adoption, PROSE treatment is available at 11 centers in the United States and 4 worldwide (October 2012, http://www.bostonsight.org/). We look forward to developing algorithms for the automated image-guided custom design of prosthetic devices for treatment of corneal disease and to generating new clinical protocols for the assessment of fit of these devices, adding image-based elements to the existing clinician and patient-based elements. We anticipate that the advances presented in this pilot study will lead to wider adoption of PROSE treatment in the care of patients with complex corneal disease.

## Figures and Tables

**Table 1:** Characteristics of PROSE devices.

		Device diameter (mm)	Haptic type
Subject 1	Eye #1	20	Spherical
Subject 2	Eye #2	18.5	Toric
Subject 3	Eye #3	18.5	Toric
Subject 3	Eye #4	18.5	3-profile
Subject 4	Eye #5	19.5	3-profile
Subject 5	Eye #6	18.5	4-profile

**Table 2:** Within-subject variance of OCT-measured parameters.

	Mean	Pooled SD	CV
Corneal diameter (mm)	13.09	0.10	0.76%
Corneal sagittal height (mm)	3.27	0.07	2.06%
Scleral sagittal height (mm)	5.14	0.17	3.39%
Scleral toricity (mm)	0.156	0.087	—
